# From Incidents to Insights: A Roadmap for Using Data on Individual Professionalism Lapses to Guide Programming and Enhance Institutional Culture

**DOI:** 10.1007/s40670-026-02898-y

**Published:** 2026-09-24

**Authors:** Stacey R. Rose, Aimee K. Gardner, Peter J. Boedeker, Ellen M. Friedman

**Affiliations:** 1 Baylor College of Medicine, One Baylor Plaza, Cullen 206A, Houston, Texas 77030 USA; 2 University of Colorado School of Medicine, Aurora, Colorado USA

**Keywords:** Professionalism competencies, Unprofessional behavio, Interpersonal communication, Quality improvement

## Abstract

While reporting and remediating unprofessional behavior is critical for patient safety and workplace collaboration, focusing on individual incidents may miss opportunities to promote professionalism at the institutional level. Here, we outline and demonstrate a five-step process for analyzing reports of unprofessional behavior at an academic medical center to inform future planning and quality improvement efforts. Drawing on our institutional experience, we detail how a structured approach (i.e. selection of a competency framework, collection and categorization of reported unprofessional behavior, data analysis, and creation of targeted programming based on identified themes) can convert individual incidents into opportunities to enhance institutional culture.

## Introduction

The importance of professionalism in medical training and practice is well established. Repeated studies have shown the negative impact professionalism lapses can have within an organization [1–3]. For example, professionalism lapses among teams can decrease learning motivation, engagement, and well-being [4–6]. Poor professionalism among clinicians also negatively impacts medical and surgical outcomes [7, 8]. Given the impact of unprofessional behavior on patient safety, institutions have a responsibility to identify and address individual professionalism lapses in order to optimize care quality and reduce patient harms [9].

Despite broad agreement regarding the importance of professionalism remediation, guidance for identifying and addressing professionalism lapses at academic medical centers is lacking [10]; indeed, a recent systematic review of professionalism remediation efforts among medical students, residents, and doctors found a dearth of evidence to guide best practices [11]. Additionally, the absence of a universally adopted definition of professionalism has been cited as an obstacle in optimizing its instruction, assessment, and remediation [12].

While most academic health centers have processes for reporting professionalism concerns, professionalism remediation efforts often focus on individual failings, without taking into account systems-level contributions [13, 14]. When a report comes in, it is treated in isolation and addressed in isolation. By contrast, a structured approach to detecting patterns in individual-level data could help institutions more effectively enhance their professional culture [9], provided the data is interpreted cautiously and with consideration of the potential for biases in incident reporting. Stated another way, reviewing professionalism lapses may aid in identifying institutional priorities, laying the groundwork for more robust, integrated and contextualized professionalism curricula [15].

While existing literature acknowledges the importance of organizational professionalism and systems-level contributions to lapses in professionalism [16, 17], there is a lack of practical guidance for how institutions can leverage individual-level reports of unprofessional behavior to drive systems-level improvements. To address this gap, we propose a five-step process for utilizing data on individual unprofessionalism reports to identify patterns that inform broader intervention, such as systems-level professionalism programming. This process grew out of an institutional review within our Center for Professionalism which has informed the subsequent selection of workshops, trainings, and invited speakers. We offer this approach as a “roadmap” for other institutions that seek to leverage individual-level data on professionalism lapses as opportunities for broader quality improvement.

## Institutional Context and Orientation towards Systems-Level Interventions and Quality Improvement

Our institution is a large, urban, academic medical center with over 4,000 full-time faculty, more than 100 accredited graduate medical education training programs, and multiple clinical affiliates [18]. The institution’s Center for Professionalism (the Center), founded in 2013, serves as a centralized resource for reporting and remediating unprofessional behavior and also promoting positive professionalism through education, training, and recognition [19–21].

As part of routine practices, the Center tracks data on reports of unprofessional behavior and remediation interventions. In 2023, as the Center completed its 10-year anniversary, the leadership sought to review existing data on individual professionalism lapses with an orientation towards systems-based initiatives to enhance the professional culture. This approach is consistent with outlined competencies for organizational professionalism, including “collective self-reflection” to “close the gap between current performance and the ideal state” [16]. More specifically, we sought to create a structured process for analyzing reports of unprofessional behavior to identify broader themes and opportunities for systems-level interventions. This process is not intended to be static; rather, the process of reflecting on professionalism lapses and designing targeted interventions can be iterative, akin to the Plan-Do-Study-Act (PDSA) framework for quality improvement [22].

In the following paragraphs, we describe and demonstrate a five-step process (Table 1) for using routinely collected professionalism-lapse reports to identify institutional priorities for professionalism programming. The process may be adapted by other institutions seeking to utilize information on individual professionalism lapses to inform quality improvement in professionalism.

**Table 1:** Five-step process for using individual reports of unprofessional behavior to inform planning for quality improvement in professionalism

Step	Description	Practical outputs
1. Selection of a Professionalism Competency Framework	Selection of an established framework to help establish a shared institutional understanding of professional behaviors	Selected framework
2. Data Collection	System for secure and consistent collection of data on individual professionalism lapses with details on the context, involved parties and behaviors	Secure database
3. Categorization of Professionalism Lapses	Individual professionalism lapses are reviewed and classified based on the selected professionalism competency framework	Coded reports
4. Data Analysis	Analysis should seek to identify themes from the data, such as frequencies and types of reported unprofessional behaviors, context and involved parties	Trend analysis
5. Creation of Targeted Institutional Programming	Based on identified trends, convene with leadership to develop strategies for quality improvement (e.g. educational initiatives) as part of Planning phase of PDSA	Targeted programming plan

## Description of Five-Step Process

### Step 1: Selection of a Professionalism Competency Framework

To facilitate a systematic analysis of reported professionalism lapses, a professionalism competency framework must be selected. By having a shared, consistent framework, data collected on each reported lapse in professionalism (Step 2) can be systematically coded and classified (Step 3). Additionally, such a framework provides a shared mental model across an institution for what counts as professional behavior. There are several frameworks that exist, many with overlapping features [23–33].

### Step 2: Data Collection

Consistent collection of incident reports is critical to facilitate later thematic analysis. Reports should capture information on context, the parties involved, and the nature of each professionalism lapse. Establishing systems for faculty, staff, trainees, etc. to submit professionalism lapses without fear of recourse is necessary. This requires providing the option for anonymity and multiple avenues by which a concern can be submitted (e.g., phone call, email, form submission with the option to exclude the submitter’s contact information). Data should be handled securely and in accordance with institutional regulations.

### Step 3: Categorization of Professionalism Lapses

Using the professionalism framework selected in Step 1, Step 3 involves categorizing each reported lapse into one or more domains. Classification can be done by individuals on a committee independently and then in duplicate with discussions to resolve disagreements until reliable coding is achieved. Through the practice of categorization and discussing discrepancies between coders, faculty and staff can develop a deeply shared mental model of professionalism. Periodic checking of classifications is recommended to ensure classification practices remain consistent.

### Step 4: Data Analysis

Analysis of the categorized incident reports can provide insights for the development of targeted interventions and institutional programming. These analyses should seek to identify themes from the data, such as the most commonly-reported types of unprofessional behaviors, or a comparison of professionalism lapses based on the context or involved parties. This can involve descriptive statistics and counts or more sophisticated statistical analyses, depending on available resources. Regardless of the methodology used, the goal is to identify areas of repeated concern that can be used to guide institutional programming (Step 5).

### Step 5: Creation of Targeted Institutional Programming

The final step in the process involves reviewing the trends and patterns which emerge from the data analysis, and developing strategies for improving institutional professionalism, based on these findings. For example, if professionalism lapses appear more frequently in a particular context or setting, then targeting education among faculty / staff / trainees in that setting may be appropriate. The creation of skill-building opportunities for a particular professionalism competency may be indicated if the data shows a high prevalence of unprofessional behavior related to that competency. It may be useful to convene with institutional leadership or to create a task force or committee to derive collaborative solutions, based on the available data. From a quality improvement perspective, this step informs the planning phase of the PDSA cycle; future analysis would be needed to determine whether the programming was effective, and to make additional modifications as necessary.

## Institutional Demonstration

In what follows, we describe how this process was followed at Baylor College of Medicine. The first four steps are outlined and then the results of the review are presented. These results then informed our decisions made in Step 5.

### Step 1: Selection of a Professionalism Competency Framework

Our institution selected the American Association of Medical Colleges (AAMC) premed professional competency framework for categorizing professionalism lapses. This choice was made based on the framework’s breadth, simplicity, congruence with comparable constructs such as the Accreditation Council for Graduate Medical Education [26], the American Board of Medical Specialties [23], and the Interprofessional Education Collaborative Core Competencies [29] as well as evidence linking preprofessional competencies with behaviors later in training and practice [34, 35]. (See Appendix 1, which maps how the AAMC domains align with other physician / professionalism standards.)

### Step 2: Data Collection

Ten years (January 2014 to October 2023) of institutional records from reported professionalism lapses were collected. Reports concerning clinical and non-clinical trainees, staff, and faculty may be submitted directly to the Center for Professionalism (via phone or email), or through an anonymous online portal (the Integrity Hotline). Of note, not all reports of unprofessional behavior are shared with the Center, as individuals may choose to use alternative options for reporting or resolving concerns (such as meeting with supervisor(s) or program directors or hospital incident report systems).

For each reported professionalism lapse, data collected included (a) lapse setting (office, classroom, laboratory, clinic, emergency center, operating room, other hospital setting, or other (e.g. social media); (b) individuals affected by the behavior (self, faculty, staff, trainee, patient, peer, subordinate, supervisor, or other, e.g. public); (c) the referred individual’s gender, department, academic rank, and specialty, such as medical (e.g., internal medicine, pediatrics), hospital-based (e.g., pathology, radiology), or surgical (e.g., general surgery, orthopedics). When known, details about the individual submitting the referral such as peer, supervisor, subordinate, trainee, staff, patient, or other (e.g. security) were also recorded. Given the sensitivity of the information in the reports, all data were collected and stored securely per institutional protocols.

### Step 3: Categorization of Professionalism Lapses

Each report of unprofessional behavior was categorized as affecting a specific, primary AAMC professional competency domain, using the definitions available at the time of the study [36]: capacity for improvement; ethical responsibility to self and others, resilience & adaptability, reliability & dependability, service orientation; social skills/oral communication; and teamwork. For each professionalism lapse, the assignment of AAMC professionalism competencies was achieved following an independent review by study authors (SRR, AKG, EMF). In the case of disagreement, discussion continued until there was a unanimous decision. Table 2 provides examples of reported professionalism lapses from our institutional dataset, and how they were categorized, using the lens of the AAMC competency framework.

**Table 2:** Institutional demonstration: examples of professionalism lapses in our institutional dataset, categorized by AAMC competency*

AAMC Premed Professional Competency (original terminology)	AAMC Premed Professional Competency (updated terminology)	Example from dataset**
Capacity for Improvement	Commitment to learning and growth	Trainee with poor insight about performance
Cultural competence	Cultural awareness / humility	Offensive post to social media
Ethical responsibility to self and others		Prioritizing personal gain over patient care
Reliability & dependability		Repeatedly late for clinical shifts
Resilience & adaptability		Yelling at colleagues during times of stress
Service orientation		Lack of sensitivity towards others’ needs
Social skills / Oral communication	Interpersonal skills / Oral communication / Empathy & compassion	Bullying communication style; speaking in a demeaning or intimidating tone
Teamwork and collaboration		Failure to coordinate vacation schedule with team

*Reports were categorized using original competency terminology; for clarity, the second column shows updated terminology based on revised AAMC Competencies

**Examples are based on actual reports, but de-identified and summarized to protect confidentiality

### Step 4: Data Analysis

All reports of professionalism lapses received by the Center for Professionalism during the designated timeframe were included for analysis, including incomplete reports and those that were submitted anonymously or confidentially; there were no exclusion criteria. Multiple referrals involving the same person were handled separately. If a report was missing data (such as information on the gender of the referred individual), this was noted and taken into account during data analysis (e.g. with adjustment of the denominator).

Our intent was to identify groups who were referred for professionalism concerns at the highest rates, the contexts in which those professionalism lapses were occurring, and which lapses occurred most frequently. Descriptive and comparative statistics were used to explore trends and compare patterns of professionalism lapses in terms of affected groups, contexts, and AAMC competencies.

### Findings from the Ten-Year Review

Results from the baseline analysis of our institutional data are presented in the following paragraphs, and in Tables 3 and 4. Between January 2014 and October 2023, the Center received 280 unique reports of unprofessional behavior; 30 individuals had multiple reports, though each report was handled separately. Reports spanned 80% (21/26) of clinical and basic science departments at the institution. Most concerns were reported by a Chair/supervisor (35%), colleague (31%), or Human Resources (21%); 3 reports were submitted anonymously (1%).

**Table 3:** Institutional demonstration: frequency of professionalism lapses in our dataset, categorized by professional competency (*N* = 280)

Capacity for Improvement (*Commitment to learning and growth)	Number of referrals	Percentage
	9	3.2%
Cultural competence (*Cultural awareness / humility)	35	12.5%
Ethical responsibility to self and others	41	14.6%
Reliability & dependability	12	4.3%
Resilience & adaptability	50	17.9%
Service orientation	4	1.4%
Social skills / Oral Communication (*Interpersonal skills / Oral communication / Empathy & compassion)	95	33.9%
Teamwork and collaboration	34	12.1%

*Updated terminology based on revised AAMC Competencies

**Table 4:** Institutional demonstration: frequency of professionalism lapses in our dataset, categorized by gender of reported individual (*N*=278^α^)

	Total Number of Referrals	Percentage & Frequency of Men’s Total Referrals (*N* = 169)	Percentage & Frequency of Women’s Total Referrals (*N* = 109)	Difference
Capacity for Improvement(*Commitment to learning and growth)	9	1% (*N* = 1)	7% (*N* = 8)	ns
Cultural competence (*Cultural awareness / humility)	35	20% (*N* = 33)	2% (*N* = 2)	*p* < 0.001
Ethical responsibility to self and others	40	15% (*N* = 25)	14% (*N* = 15)	ns
Reliability & dependability	12	4% (*N* = 7)	4% (*N* = 5)	ns
Resilience & adaptability	49	20% (*N* = 34)	14% (*N* = 15)	*p* < 0.05
Service orientation	4	2% (*N* = 3)	1% (*N* = 1)	ns
Social skills / Oral communication (*Interpersonal skills / Oral communication / Empathy & compassion)	95	29% (*N* = 49)	42% (*N* = 46)	*p* < 0.01
Teamwork and collaboration	36	10% (*N* = 18)	16% (*N* = 18)	ns

α *N* = 278 reports for which gender data was available

b differences were explored using independent samples t-test

*Updated terminology based on revised AAMC Competencies

Almost half of the reported professionalism lapses occurred in the inpatient hospital (26%) and clinic (23%) settings, with the remaining professionalism lapses occurring in the operating room (14%), office (11%), classroom (7%), emergency room (7%), laboratory (5%), or other (7%). The academic rank of faculty reported for unprofessional behavior was evenly distributed (34% Assistant Professors; 32% Associate Professors; 33% Professors); for comparison, only 14% of full-time faculty at our institution are at the rank of Associate Professor or higher [unpublished institutional data, 2024].

Table 3 depicts the frequency of professionalism lapses by AAMC competency; most reported concerns were linked to competency gaps in social skills/oral communication (33.9%) and resilience & adaptability (17.9%), followed by ethical responsibility (14.6%), cultural competence (12.5%), and teamwork (12.1%).

Reports involving men accounted for more than half of all reported lapses (*N* = 169 or 60%). As shown in Table 4, reports involving men more often described professionalism lapses in the domains of cultural competency (20% of reports among men versus 2% of reports among women, *p* < 0.001) and resilience & adaptability (20% of reports among men versus 14% of reports among women, *p* < 0.05). By contrast, reports involving women were more likely to describe professionalism lapses related to social skills/oral communication (42% of reports among women versus 29% of reports among men, *p* < 0.01). Notably, without denominators such as total faculty by gender, this data merits cautious interpretation. Additionally, reports of professionalism lapses may not represent the true prevalence of unprofessional behavior, since reporting may be influenced by awareness, reporting culture, power dynamics, institutional trust, specialty norms, gender expectations and accessibility of reporting mechanisms. The interpretation of gender differences in reported lapses in professionalism merits particular caution, given known biases – for example, women may be judged differently than men in relation to social communication [37]. Nevertheless, the reports provide a window into potential opportunities for systems-level interventions to advance professional behaviors.

### Step 5: Creation of Targeted Institutional Programming

At our institution, following a review of the data trends and patterns, the leadership of the Center for Professionalism and collaborators (Office of Faculty Development; Office of Community Engagement; Center for Medical Ethics and Health Policy) created a plan for targeted programming in alignment with several of the identified challenges. Table 5 details several of the implemented interventions, which ranged from a professionalism skill-building session for graduating medical students, to departmental and institution-wide educational series. For example, the preponderance of reports of professionalism lapses related to the AAMC domain of cultural competence led to the inclusion of teaching on “affinity bias” as part of a one-hour presentation (“Professionalism Matters”) offered to clinical and basic science departments. Follow-up analyses – such as participation in programming, longitudinal follow up surveys among participants of educational interventions, as well as institution-level data such as learning environment surveys and ACGME milestone data, are planned to determine the effectiveness of these interventions in addressing professionalism challenges and enhancing institutional culture. In keeping with the PDSA framework, the Center has also begun to prospectively categorize professionalism lapses by AAMC competency and will review trends on an annual basis.

**Table 5:** Institutional demonstration: examples of interventions to promote professionalism in alignment with identified trends in reported unprofessional incidents

Identified Trend	Intervention	Target Audience	Implementation Status	Implemented or Planned Evaluation / outcome
Preponderance of reports of professionalism lapses related to AAMC competency gaps in: -social skills / oral communication -resilience and adaptability -cultural competence	Development of a 1-hr presentation (“Professionalism Matters”) - offered to all clinical and basic science departments – to promote a shared understanding of professionalism expectations, including practical strategies related to communication and cultural humility	Faculty, learners and staff across all departments	Delivered to 17/18 clinical departments and 4/7 basic science departments, typically as part of Grand Rounds	In post-session surveys, participants report increased understanding of professionalism and intention to change behavior (manuscript under preparation)
	Development of a case-based workshop highlighting commonly-encountered professionalism challenges during residency including themes related to communication, resilience / adaptability and cultural competence	Graduating medical students, with faculty and near-peer facilitators	Completed 2 sessions as part of Transition to Residency Course (~ 170 students at each session)	Post-session surveys show improved participant comfort in managing professionalism challenges; planning additional, longitudinal follow up to determine whether learned strategies have been implemented during residency (manuscript under review)
	Creation of a series of 5-min, case-based professionalism teachings (“Professionalism in Practice”) which can be added to the beginning of an existing conference (such as grand rounds); each case aligns with a AAMC professionalism competency and uses a consistent framework for navigating professionalism challenges	Faculty, learners and staff across multiple departments	Implemented as part of Grand Rounds and/or faculty meeting(s) in Internal Medicine, Surgery, Pediatric Hospital Medicine, Family and Community Medicine, Obstetrics and Gynecology (47 sessions to date)	In post session surveys, participants report that cases feel relevant to their day-to-day practice, and that the PiP framework will be useful in approaching future professionalism challenges (see published report, PMID 42137404)
Gender differences in reported professionalism lapses	Selection of speakers for the Center for Professionalism’s quarterly series (“Profiles in Professionalism”) with expertise in cultural and gender disparities in medicine	Faculty, learners and staff across the institution	Invited speakers (2025–2026) have discussed topics related to disparities in health care policy, resilience in science and medicine, and self-care	Favorable post-session surveys and unsolicited feedback from participants; plan to engage with undergraduate and graduate medical education leaders to monitor gender / cultural themes in institutional learning environment reports
	Implementation of a “town hall” discussion on professionalism as a forum to discuss different perspectives / encountered bias	Faculty, learners and staff across the institution	In March 2026,the Center hosted “Professionalism, It’s Complicated” – a panel discussion / town hall open to faculty, learners and staff across the institution	146 participants engaged in discussion at the event; BCM Professionalism Partners are now collaborating on an interactive didactic session on “vantage points in professionalism” to be offered to all departments (and will include post-session surveys)

### Generalizability of the Five-Step Process: Lessons Learned and Future Directions

The identification and remediation of individuals with unprofessional behavior are critical elements of promoting a culture of professionalism [1, 38]. At the same time, focusing on individual incidents of unprofessional behavior may miss the opportunity to detect systems-level contributions, or to promote an organizational culture of professionalism, such as through institution-wide programming [9, 13, 14].

This report outlines a process followed to determine local professionalism needs to guide selection of institution-level professionalism programming. Given that the majority of academic health centers include a system for reporting unprofessional behavior, this work may serve as a roadmap for other institutions seeking to turn individual incidents into opportunities for broader improvement in professional behaviors.

We selected the AAMC premed professional competencies for Step 1 (selection of a professionalism competency framework). While the AAMC framework was designed for entering medical students, the described competencies are clear and succinct, and are well aligned with professionalism standards developed for physicians and other health science professionals [23–33] (Appendix 1). An alternative framework may appeal to other institutions seeking to engage in a structured review of professionalism lapses; for example, if a residency program is seeking to review its data, using the Accreditation Council for Graduate Medical Education professionalism milestones may be more germane. Regardless of the selected framework, our findings suggest that there is utility to organizing individual lapses in professionalism by competency; this approach helps to facilitate the identification of competency gaps to inform future programming.

For Step 2 (data collection), we focused on professionalism lapses reported to the institution’s Center for Professionalism, which maintains confidential data on all reported concerns. Other institutions may seek alternative mechanisms for data collection; for instance, prior studies have analyzed unprofessional behavior as reported through student [39] or faculty [40] evaluations. However, to identify trends at a systems-level, it may be necessary to routinely collect data on the referred individual (e.g. role; academic rank, gender, medical specialty) as well as the location / context in which the unprofessional behavior was observed. Notably, in our dataset, there was a disproportionate representation of higher-ranked faculty among those reported for professionalism lapses; this finding underscores the importance of confidential reporting mechanisms to support more vulnerable members of the academic health center community, such as trainees and junior faculty, so that accurate data on professionalism lapses can be collected [41].

For Step 3 (categorization of professionalism lapses), we found it helpful to use multiple reviewers who could then discuss / adjudicate any discrepancies in the classification. While our process for categorization involved a retrospective review of collected incident reports, it may be advisable to consider strategies for prospective categorization. Indeed, as of January 2026, the Center has a process for categorizing each professionalism lapse by competency at the time of the report.

For Step 4 (data analysis), we used descriptive statistics to identify trends, such as the professionalism competency gaps most frequently reported, their context(s), and characteristics of the individual with the reported professionalism lapse (e.g. academic rank, gender). Other institutions may choose to analyze the data differently, such as employing qualitative thematic analysis, or grouping the data based on the type of reporter (faculty, staff or trainee). Consistent with the bidirectional nature of quality improvement processes, data analysis (Step 4) may shed light on ways to improve data collection (Step 2). For example, in our dataset, there was a high preponderance of reports related to challenges with the AAMC professional competency of resilience & adaptability. Given that a lack of resilience & adaptability may relate to underlying burnout and associated systems-level challenges [42], our Center (as of January 2026) has begun collecting information on systems-level contributions to each reported professionalism concern. This additional information may inform future analyses and quality improvement interventions, such as identifying and addressing institutional issues.

While the initial baseline review analyzed ten years of institutional data, moving forward, the data will be reviewed annually to identify emerging trends and inform annual programming for the Center. Other institutions may elect to analyze collected data more or less frequently, as appropriate for their setting(s).

As part of Step 5 (creation of targeted institutional programming), we used the observed trends in the dataset to inform the development of educational and other initiatives. As the reports of unprofessional behavior spanned all roles and academic ranks, interventions were directed to a wide range of audiences, including trainees, residents and faculty. Additionally, given the breadth and complexity of our academic health center, we found it useful to convene with other institutional leaders (such as the Office of Faculty Development, the Office of Community Engagement, and the Center for Medical Ethics and Health Policy) to identify shared goals and opportunities for synergy. Such an approach would likely be applicable to other institutions with a similarly complex structure and resource limitations.

While the five-step process may be more difficult for institutions without a centralized professionalism center to implement, the approach could be adapted on a smaller scale; for example, an individual department could establish a framework for professionalism competencies and set up an anonymous, electronic reporting system for professionalism concerns including data on the reported individual (gender, academic rank), the location / context of the incident (inpatient, outpatient, classroom), and a description of the incident which could then be categorized based on the selected competency framework. The “roadmap” as described is intended to be modifiable based on institutional priorities and resources.

### Limitations

The proposed five-step process – aiming to turn individual reports of unprofessional behavior into opportunities for institutional improvement – has several limitations worth noting. As we implemented it, the process is resource-intensive, requiring a dedicated institutional process for collecting and analyzing reported professionalism lapses. For our institution, the Center for Professionalism is well positioned for this role, with existing mechanisms for consistent and secure collection of reported concerns. While many academic medical centers have similar centralized services for reporting and addressing professionalism concerns, the five-step process may need to be modified or scaled based on available institutional procedures and resources.

Even with a centralized resource for reporting professionalism lapses, there can be challenges with underreporting, or the inability to capture unprofessional behavior that is managed (informally or formally) by other individuals or entities outside the Center for Professionalism (such as departmental leaders or Human Resources). Additionally, we did not collect data on interrater reliability for coding professionalism lapses by AAMC competency, which may limit the reproducibility of the findings. We have subsequently revised our data collection system to include categorizing the professionalism lapse at the time of the report (rather than retrospectively), which may improve accuracy and allow for an assessment of interrater reliability. Nevertheless, despite using a structured approach - our analyses may not completely portray the professionalism competency gaps at our institution. Similar limitations would likely apply to other institutions seeking to implement the proposed five-step process.

Finally, reports of unprofessional behavior may be impacted by individual and social biases; for instance, in our institutional dataset, gender differences appeared to emerge, both in the number and nature of reported professionalism concerns. Specifically, 60% of the individuals referred for concerns were men, compared to a faculty composition of only 45% men (unpublished institutional data, 2024). These data should be interpreted cautiously given the lack of denominators such as total faculty by gender; however, the findings are in line with previous reports in which men were more frequently considered the source of unprofessional behavior [6, 43, 44], while women experience more discrimination and microaggressions than their male counterparts during training and practice [45, 46]. Additionally, in our dataset, reports involving women were more likely to pertain to unprofessional behavior in the domains of social skills/oral communication. Prior studies have shown that women are expected to be more socially sensitive compared to their male counterparts [37]. Similar biases have been reported based on medical specialties [47, 48], which may impact the interpretation of patterns of reporting of unprofessional behavior by specialty or context. Consequently, the analysis of reports of unprofessional behavior, and the creation of targeted institutional programming, should acknowledge the contributions of differential expectations and social biases [49].

## Conclusions

In summary, we propose a structured five-step process to turn individual reports of professionalism lapses into opportunities to enhance professionalism programming across the institution, using our institutional experience as a demonstration.

Our approach may serve as a roadmap for other academic medical centers, as routinely collected data on professionalism lapses may help institutions identify professionalism competency gaps and guide targeted programming as part of an iterative quality-improvement cycle.

Future work is needed to evaluate whether targeted programming changes, based on identified trends in reports of unprofessional behavior, lead to meaningful changes in professionalism, as measured by reporting patterns of professionalism lapses and other indicators of the professional climate, such as ACGME milestones and learning environment surveys.

## Data Availability

The institutional datasets generated and analyzed for this work are not publicly available, due to their sensitivity and per institutional policy. Additional information may be available from the corresponding author on a case by case basis, per reasonable request.

## Appendix 1

**Table 6:** Distribution of common professionalism competencies across professionalism constructs in the health professions

Professionalism construct	Representation of Common Professionalism Competencies									
	Commitment to Growth	Cultural Awareness	Self-Reflection	Accountability & Conscientiousness	Ethical Behavior	Respect for Others	Team Collaboration	Compassion & Empathy	Reliability & Dependability	Communication Skills
AAMC Premed Professional Competencies	X	X	X	X	X	X	X	X	X	X
ACGME Milestones	X	(implicit)	X	X	X	X	(implicit)	X	X	(implicit)
AACN: The Essentials	X	X	X	X	X	X	(implicit)	X	X	X
ABMS Position Statement on Professionalism	X	X	X	X	X	X	(implicit)	X	X	X
ABIM Physician Charter	X	X	(implicit)	X	X	X	X	X	X	X
AOA Medical Professionalism: Best Practices	X	X	X	X	X	X	(implicit)	X	X	X
ASCLS “Beyond the Lab Coat”	X	X	X	X	X	X	X	X	X	X
ASAHP Position Statement on Interprofessional Education	X	X	(implicit)	X	(implicit)	X	X	(implicit)	(implicit)	X
IPEC Core Competencies	X	S	X	X	X	X	X	X	X	X
The Core Entrustable Professional Activities (EPAs) for Entering Residency	X	(implicit)	X	X	X	X	X	X	X	X
AMA Principles of Medical Ethics	X	(implicit)	X	X	X	X	X	X	X	X

## References

[1] Brennan MD, Monson V. Professionalism: good for patients and health care organizations. Mayo Clin Proc. 2014;89(5):644–52. 10.1016/j.mayocp.2014.01.011 24797645

[2] Roberts NK, Dorsey JK, Wold B. Unprofessional behavior by specialty: a qualitative analysis of six years of student perceptions of medical school faculty. Med Teach. 2014;36(7):621–5. 10.3109/0142159X.2014.899690. 24787525

[3] Owens K, Eggers J, Keller S, McDonald A. The imperative of culture: a quantitative analysis of the impact of culture on workforce engagement, patient experience, physician engagement, value-based purchasing, and turnover. J Healthc Leadersh. 2017;9:25–31. 126381. 10.2147/JHL.S126381. PMC5774450 29355220

[4] Westbrook J, Sunderland N, Li L, Koyama A, McMullan R, Urwin R, et al. The prevalence and impact of unprofessional behaviour among hospital workers: a survey in seven Australian hospitals. Med J Aust. 2021;214(1):31–7. 10.5694/mja2.50849. 33174226

[5] Gan R, Snell L. When the learning environment is suboptimal: exploring medical students’ perceptions of mistreatment. Acad Med J Assoc Am Med Coll. 2014;89(4):608–17. 10.1097/ACM.0000000000000172 PMC4885564 24556767

[6] Rowe SG, Stewart MT, Van Horne S, Pierre C, Wang H, Manukyan M, et al. Mistreatment experiences, protective workplace systems, and occupational distress in physicians. JAMA Netw Open. 2022;5(5):e2210768. 10.1001/jamanetworkopen.2022.10768. PMC9077480 35522279

[7] Cooper WO, Guillamondegui O, Hines OJ, Hultman CS, Kelz RR, Shen P, et al. Use of unsolicited patient observations to identify surgeons with increased risk for postoperative complications. JAMA Surg. 2017;152(6):522–9. 10.1001/jamasurg.2016.5703. PMC5831421 28199477

[8] Cooper WO, Spain DA, Guillamondegui O, Kelz RR, Domenico HJ, Hopkins J, et al. Association of coworker reports about unprofessional behavior by surgeons with surgical complications in their patients. JAMA Surg. 2019;154(9):828–34. 10.1001/jamasurg.2019.1738. PMC6585020 31215973

[9] Martinez W. From insight to action: tackling underperformance in health professionals. BMJ Qual Saf. 2025;34(2):77–80. 10.1136/bmjqs-2024-017682. 39500556

[10] Hauer KE, Ciccone A, Henzel TR, Katsufrakis P, Miller SH, Norcross WA, et al. Remediation of the deficiencies of physicians across the continuum from medical school to practice: a thematic review of the literature. Acad Med. 2009;84(12):1822–32. 10.1097/ACM.0b013e3181bf3170. 19940595

[11] Brennan N, Price T, Archer J, Brett J. Remediating professionalism lapses in medical students and doctors: a systematic review. Med Educ. 2020;54(3):196–204. 10.1111/medu.14016. 31872509

[12] Nuts and Bolts of Professionalism Remediation. In: Remediation in Medical Education [Internet]. Cham: Springer International Publishing. 2023 [cited 2025 Jul 18]. pp. 163–81. Available from: https://link.springer.com/10.1007/978-3-031-32404-8_14

[13] Ziring D, Danoff D, Grosseman S, Langer D, Esposito A, Jan MK, et al. How do medical schools identify and remediate professionalism lapses in medical students? a study of U.S. and Canadian medical schools. Acad Med. 2015;90(7):913–20. 10.1097/ACM.0000000000000737. 25922920

[14] Kalet A, Chou CL, Ellaway RH. To fail is human: remediating remediation in medical education. Perspect Med Educ. 2017;6(6):418–24. 10.1007/S40037-017-0385-6. PMC5732108 29071550

[15] O’Sullivan H, Van Mook W, Fewtrell R, Wass V. Integrating professionalism into the curriculum: AMEE Guide 61. Med Teach. 2012;34(2):e64–77. 10.3109/0142159X.2012.655610. 22288994

[16] Egener B, McDonald W, Rosof B, Gullen D. Perspective: organizational professionalism. Acad Med. 2012;87(5):668–74. 10.1097/ACM.0b013e31824d4b67. 22450182

[17] Carroll MR, Barak G, Byrne T, Farid H. Promoting professionalism in medical education: a toolbox for effective feedback and remediation. Clin Teach. 2025;22(6):e70244. 10.1111/tct.70244. PMC12614289 41231517

[18] Baylor College of Medicine. About Us [Internet]. [cited 2025 Jul 18]. Available from: https://www.bcm.edu/about-us

[19] Rose S, Kusnoor A, Huynh P, Greely J, Rojas-Khalil Y, Kung D, et al. Addressing unprofessional behaviors in the clinical learning environment: lessons from a multi-year virtual, intergenerational, interdisciplinary workshop. Med Educ Online. 2024;29(1):2316491. 10.1080/10872981.2024.2316491. PMC10868425 38354128

[20] Tomlinson M, Nasto K, Gosselin K, et al. Fruits of the Professional Educator Appreciation and Recognition (PEAR) awards: learning what students value in their medical educators. Med Sci Educ. 2025;35:787–91. 10.1007/s40670-024-02234-2. PMC12058585 40352999

[21] Godwin KM, Ali A, Laufman L, Friedman EM. A Multipronged effort to incorporate professionalism into campuswide programming through a center for professionalism. J Grad Med Educ. 2025;17(2):229–34. 10.4300/JGME-D-24-00663.1. PMC12096131 40417106

[22] Taylor MJ, McNicholas C, Nicolay C, Darzi A, Bell D, Reed JE. Systematic review of the application of the plan–do–study–act method to improve quality in healthcare. BMJ Qual Saf. 2014;23(4):290–8. 10.1136/bmjqs-2013-001862. PMC3963536 24025320

[23] ABMS Position Statement on Promoting Professionalism [Internet]. 2024 [cited 2025 Jan 22]. Available from: https://www.abms.org/newsroom/abms-position-statement-on-promoting-professionalism/

[24] AMA Principles of Medical Ethics [Internet]. [cited 2025 Jul 18]. Available from: https://code-medical-ethics.ama-assn.org/principles

[25] ASAHP Position Statement on Interprofessional Education [Internet]. [cited 2025 Jul 17]. Available from: https://www.asahp.org/ipe

[26] Edgar L, Roberts S, Holmboe E. Milestones 2.0: a step forward. J Grad Med Educ. 2018;10(3):367–9. 10.4300/JGME-D-18-00372.1 PMC6008021 29946411

[27] The Premed Competencies for Entering Medical Students [Internet]. 2021 [cited 2025 Jan 22]. Available from: https://students-residents.aamc.org/real-stories-demonstrating-premed-competencies/premed-competencies-entering-medical-students

[28] Medical Professionalism Best Practices. Professionalism in a Relentless World [Internet]. Alpha Omega Alpha Honor Medical Society; [cited 2025 Jul 18]. Available from: https://www.alphaomegaalpha.org/wp-content/uploads/2025/02/Final-with-cover-2024-Professionalism-Monograph.pdf

[29] IPEC Core Competencies for Interprofessional Collaborative Practice. Version 3 [Internet]. 2023 [cited 2025 Jul 18]. Available from: https://bit.ly/IPEC-core-competencies-v3

[30] Ingham P. Crafting Character and Cultivating Professionalism in Aspiring Medical Laboratory Scientists and Technicians [Internet]. American Society for Clinical Laboratory Science; 2023 Dec [cited 2025 Jul 17]. Available from: https://ascls.org/beyond-the-lab-coat/

[31] Foundation ABIM, Foundation ACP-ASIM. European Federation of Internal Medicine. Medical professionalism in the new millennium: a physician charter. Ann Intern Med. 2002;136(3):243–6. 10.7326/0003-4819-136-3-200202050-00012. 11827500

[32] American Association of Colleges of Nursing (AACN). The Essentials, Domain 9: Professionalism [Internet]. [cited 2025 Jul 18]. Available from: https://www.aacnnursing.org/essentials/tool-kit/domains-concepts/professionalism

[33] The Core Entrustable Professional Activities. (EPAs) for Entering Residency [Internet]. [cited 2025 Jul 18]. Available from: https://www.aamc.org/about-us/mission-areas/medical-education/cbme/core-epas

[34] Wasserman JA, Lopez AA, Knickerbocker R, Haywood A, Kibble J, Fraser R. What premedical students need to succeed: updated premed competencies for entering medical students. Acad Med. 2025;100(12):1452–8. 10.1097/acm.0000000000006065. 40249857

[35] Ballejos MP, Cestone C, Copeland HL, Dunleavy DM, Geiger T, Patel D. Predicting medical student performance with a situational judgment test in admissions. Acad Med. 2024;99(2):175–82. 10.1097/acm.0000000000005516. 37976374

[36] The Core Competencies for Entering Medical Students. Preprofessional Competencies [Internet]. [cited 2025 Jul 18]. Available from: https://students-residents.aamc.org/media/15376/download?attachment

[37] Hentschel T, Heilman ME, Peus CV. The multiple dimensions of gender stereotypes: a current look at men’s and women’s characterizations of others and themselves. Front Psychol. 2019;10:11. 10.3389/fpsyg.2019.00011. PMC6364132 30761032

[38] Egener BE, Mason DJ, McDonald WJ, Okun S, Gaines ME, Fleming DA, et al. The charter on professionalism for health care organizations. Acad Med J Assoc Am Med Coll. 2017;92(8):1091–9. 10.1097/ACM.0000000000001561. PMC5526430 28079726

[39] Mak-van der Vossen MC, van Mook WNKA, Kors JM, van Wieringen WN, Peerdeman SM, Croiset G, et al. Distinguishing Three Unprofessional Behavior Profiles of Medical Students Using Latent Class Analysis. Acad Med J Assoc Am Med Coll. 2016;91(9):1276–83. 10.1097/ACM.0000000000001206. 27119326

[40] Heath JK, Clancy CB, Dine CJ, Pluta WJS, Kogan JR. Analysis of professionalism themes raised in evaluations of faculty. JAMA Netw Open. 2022;5(7):e2221613. 10.1001/jamanetworkopen.2022.21613. PMC9280398 35816317

[41] Gill AC, Nelson EA, Mian AI, Raphael JL, Rowley DR, Mcguire AL. Responding to moderate breaches in professionalism: an intervention for medical students. Med Teach. 2015;37(2):136–9. 10.3109/0142159X.2014.911270. PMC4229483 24819504

[42] West CP, Dyrbye LN, Shanafelt TD. Physician burnout: contributors, consequences and solutions. J Intern Med. 2018;283(6):516–29. 10.1111/joim.12752. 29505159

[43] Leitman IM, Muller D, Miller S, Hanss BG, Catron TF, Cooper WO, et al. Implementation of an online reporting system to identify unprofessional behaviors and mistreatment directed at trainees at an Academic Medical Center. JAMA Netw Open. 2022;5(12):e2244661. 10.1001/jamanetworkopen.2022.44661. PMC9719046 36459140

[44] Iyer MS, Way DP, MacDowell DJ, Overholser BM, Spector ND, Jagsi R. Bullying in academic medicine: experiences of women physician leaders. Acad Med J Assoc Am Med Coll. 2023;98(2):255–63. 10.1097/ACM.0000000000005003. 36484542

[45] Heisler CA, Godecker AL, Verran D, Sinha MS, Byam J, Miller P. Workplace microaggressions: results of a survey of the American College of Surgeons members. Am J Obstet Gynecol. 2024;231(2):265.e1-265.e8. 10.1016/j.ajog.2024.04.044. 38710272

[46] Sudol NT, Guaderrama NM, Honsberger P, Weiss J, Li Q, Whitcomb EL. Prevalence and nature of sexist and racial/ethnic microaggressions against surgeons and anesthesiologists. JAMA Surg. 2021;156(5):e210265. 10.1001/jamasurg.2021.0265. PMC7992024 33760000

[47] Harendza S, Pyra M. Just fun or a prejudice? - physician stereotypes in common jokes and their attribution to medical specialties by undergraduate medical students. BMC Med Educ. 2017;17(1):128. 10.1186/s12909-017-0964-6. PMC5530564 28747204

[48] Bellodi PL. The general practitioner and the surgeon: stereotypes and medical specialties. Rev Hosp Clin. 2004;59(1):15–24. 10.1590/s0041-87812004000100004. 15029281

[49] Maristany D, Hauer KE, Leep Hunderfund AN, Elks ML, Bullock JL, Kumbamu A, et al. The problem and power of professionalism: a critical analysis of medical students’ and residents’ perspectives and experiences of professionalism. Acad Med J Assoc Am Med Coll. 2023;98(11S):S32–41. 10.1097/ACM.0000000000005367. 37983394

